# Expression of merlin, NDRG2, ERBB2, and c-MYC in meningiomas: relationship with tumor grade and recurrence

**DOI:** 10.1590/1414-431X20155125

**Published:** 2016-03-18

**Authors:** B.R. Ongaratti, C.B.O. Silva, G. Trott, T. Haag, C.G.S. Leães, N.P. Ferreira, M.C. Oliveira, J.F.S. Pereira-Lima

**Affiliations:** 1 Programa de Pós-Graduação em Patologia, Universidade Federal de Ciências da Saúde de Porto Alegre, Porto Alegre, RS, Brasil; 2 Centro de Neuroendocrinologia, Irmandade Santa Casa de Misericórdia de Porto Alegre, Porto Alegre, RS, Brasil

**Keywords:** Meningioma, Merlin, NDRG2, ERBB2, c-MYC, Immunohistochemistry

## Abstract

Meningiomas are common, usually benign tumors of the central nervous system that have a high rate of post-surgical recurrence or regrowth. We determined expression of the proteins merlin, NDRG2, ERBB2, and c-MYC in meningiomas using immunohistochemistry and assessed relationships between protein expression and gender, age, tumor grade, and recurrence or regrowth. The study sample comprised 60 patients, (44 women and 16 men) with a mean age of 53.2±12.7 years. Tumors were classified as grade I (n=48) or grades II and III (n=12). Expression of merlin, NDRG2, ERBB2, and c-MYC was not significantly different statistically with relation to gender, age, or meningioma recurrence or regrowth. Merlin was expressed in 100% of the cases. No statistically significant difference between tumor grade and recurrence or regrowth was identified. Statistically significant differences were identified between the mean age of patients with grade I (54.83±11.60) and grades II and III (46.58±15.08) meningiomas (P=0.043), between strong c-MYC expression and grades II and III (P<0.001), and between partial surgical resection and tumor recurrence or regrowth (P<0.001). These findings reveal the lower mean age among grades II and III meningioma patients than grade I patients, the influence of the protein merlin on tumorigenesis, the association of c-MYC with aggressive meningiomas, and that partial surgical resection is associated with tumor recurrence or regrowth.

## Introduction

Meningiomas represent approximately 35% of primary central nervous system (CNS) tumors, with an incidence of 7.44 cases for every 100,000 inhabitants in the United States (1). Meningiomas can develop in patients at any age, although they are more common among those in their 60s and 70s. Women are three times more likely to develop meningiomas than men (2). The World Health Organization (WHO) classifies meningiomas into three groups: benign (grade I), atypical (grade II), and anaplastic (grade III) (2). Surgery is effective in treating most meningiomas. However, some patients have inoperable, invasive, recurring, or malignant tumors; these often require alternative therapies to resection (3).

The *NF2* gene is located on the long arm of chromosome 22, which is a region commonly involved in meningioma tumorigenesis. Merlin, the product of*NF2*, acts as a tumor suppressor and its deficiency or absence is the most commonly identified genetic alteration in meningiomas (4). The *NDRG2* gene is involved in cell differentiation and tumor suppression; partial or complete loss of*NDRG2* expression is observed in several aggressive tumors (5). Furthermore, ERBB2 is a transmembrane receptor protein with tyrosine kinase activity and is involved in proliferation, differentiation, migration, adhesion, and apoptosis (6). Additionally, underexpression of ERBB2 is significantly associated with meningioma recurrence following surgical resection (7). The protein c-MYC is involved in growth regulation and cellular metabolism, and c-MYC mutations contribute to cancer development. Overexpression of c-MYC has been observed in glioblastomas, medulloblastomas, and atypical and anaplastic meningiomas (8 9 10). We have investigated the expression of merlin, NDRG2, ERBB2, and c-MYC in patient meningioma specimens using immunohistochemistry (IHC). We investigated relationships between protein expression and gender, age, tumor grade, and recurrence or regrowth to better define the factors that contribute to meningioma tumorigenesis, aggressiveness, and recurrence.

## Material and Methods

Tumor tissue samples were obtained from patients with an anatomopathological diagnosis of meningioma who underwent surgical resection by the same surgeon (N.P.F.) at Hospital São José in the Complexo Hospitalar Santa Casa de Porto Alegre between June 2013 and January 2015. The tumors were classified according to the histological criteria of the WHO regarding their subtypes and grades (11). Patient records provided clinical data including age, gender, type of surgical resection, and survival free from recurrence or regrowth. Recurrence was considered to be the reappearance of the tumor after total macroscopic resection; regrowth was considered to be the enlargement of the tumor after partial macroscopic resection (7). The study was approved by the Ethics Committee of Irmandade Santa Casa de Misericórdia de Porto Alegre and Universidade Federal de Ciências da Saúde de Porto Alegre (CAAE protocol #12559313.2.0000.5335) and was conducted in compliance with the Declaration of Helsinki. All patients gave written informed consent prior to inclusion in the study and their anonymity was preserved.

For immunohistochemical analysis, tumor tissue samples were fixed in 10% buffered formalin and embedded in paraffin. Blocks were sectioned at 4 µm, deparaffinized, and rehydrated. Antigen recovery for merlin and NDRG2 was performed using sodium citrate, pH 6.0, while Tris-EDTA, pH 9.0 was used for ERBB2 and c-MYC. Endogenous peroxidase was blocked using 5% hydrogen peroxide in methanol. A 5% skim milk in PBS solution was used to prevent nonspecific interactions. The polymer system method (Advance™ HRP Enzyme; Dako, USA) was applied to detect merlin (anti-NF2/merlin polyclonal antibody, 1:400 dilution; Abcam, USA; catalog No. ab30329), NDRG2 (anti-NDRG2 polyclonal antibody, 1:100 dilution; Santa Cruz Biotechnology, USA; catalog No. sc-50345), ERBB2 (anti-ERBB2 polyclonal antibody, clone CB11, 1:200 dilution; Novocastra Laboratories, UK; catalog No. NCL-CB11), and c-MYC (anti-c-MYC polyclonal antibody, clone Y69, 1:30 dilution; Biocare Medical, USA; catalog No. CME415AK). The primary antibody was replaced with saline solution as a negative control. Breast cancer tissue sections were used as positive controls for merlin and ERBB2 expression, salivary gland cancer sections for NDRG2, and small-cell lung carcinoma sections for c-MYC.

The presence of signals indicating merlin (12), NDRG2 (13), ERBB2 (14) (weak, moderate or strong) expression in the cytoplasm and c-MYC (15) (weak or strong) expression in the nucleus or perinucleus were considered positive. Slides were assessed by two independent and blinded observers (B.R.O. and G.T.) using a light microscope.

Results are reported as means and standard deviation for continuous variables and relative frequency and percentage for categorical variables. Associations were analyzed using a chi-squared test and, when needed, Fisher’s exact test. The Kaplan-Meier method was used to estimate the survival free from recurrence or regrowth. The statistical significance adopted was 5%. Statistical analyses were performed using SPSS version 22.0 (IBM Corp., USA).

## Results

Of the 60 patients, 44 (73.3%) were women and the mean age was 53.2±12.7 years. According to the WHO classification, 48 cases (80%) were grade I and 12 (20%) were grades II and III. The mean age of grades II and III meningioma patients was statistically lower compared with that of grade I patients (46.58±15.08*vs* 54.83±11.60; P=0.043). Table 1 provides information regarding the histological subtypes identified.

**Table:**
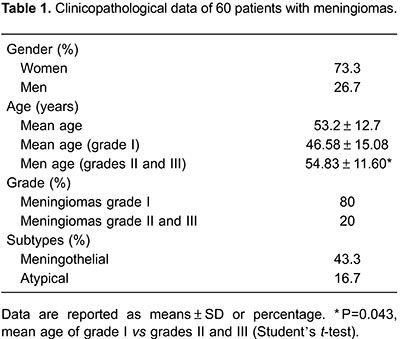

All samples were positive for merlin expression, with 40% classified as low-moderate and 60% as strong. NDRG2 expression was positive in 93.3% of samples, with 51.6% weak, 35% moderate, and 6.7% strong. ERBB2 expression was positive in 88.3% of the meningiomas studied, with 31.7% weak, 38.3% moderate, and 18.3% strong. c-MYC expression was positive in 38.3% of the cases, with 11.7% weak, and 26.6% strong. A significant difference in the percentages of high grade (II and III) and low grade meningiomas exhibiting a strong level of c-MYC expression was identified (P<0.001). No statistically significant differences were found between tumor grade and expression of merlin, NDRG2, or ERBB2. No relationships between the expression of merlin, NDRG2, ERBB2, or c-MYC and gender, age, recurrence, or regrowth were identified (Table 2).

**Table:**
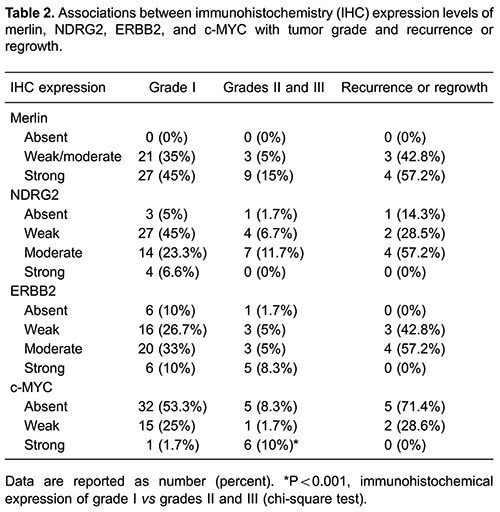

**Figure 1:**
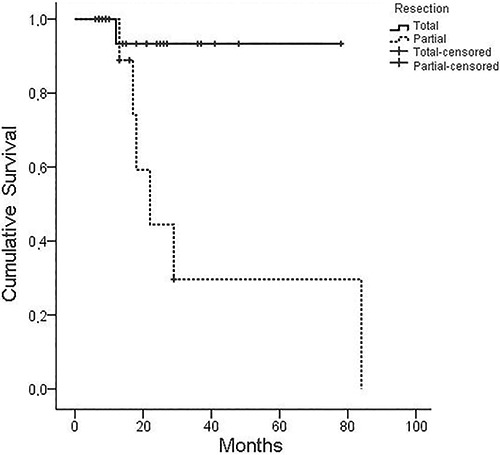
Kaplan-Meier curve of recurrence-free survival following surgical resection of meningioma (P=0.012).

Of the 60 patients in the sample, 38 were followed for an average of 22.9 months (6-84 months). In this time, two meningioma-associated deaths were reported during the perioperative period, and three additional deaths were unrelated to meningioma. The disease remained stable in 26 patients (68.4%), while seven (18.4%) had recurrence or regrowth. Of the patients with recurrence or regrowth, six had undergone partial surgical resection and one had undergone total surgical resection. Five of the lesions from patients with recurrence or regrowth were grade I, one was grade II, and one was grade III. Tumor grade was not associated with tumor recurrence or regrowth. However, partial tumor surgical resection was significantly associated with recurrence or regrowth (P<0.001). Survival free from recurrence or regrowth was 95.8% over 1 year, 75.3% over 2 years, and 65.8% over 3 years (Figure 1). It was not possible to estimate survival free from recurrence or regrowth in patients with c-MYC expression because of the low number of recurrences.

## Discussion

Meningiomas are the most common primary tumor of the CNS, and are more prevalent in women, with incidence increasing with age (1). Our patient cohort had a mean age of 53.2±12.7 years with 73.3% females, consistent with previous studies (2,14). Patients with grades II and III meningiomas had a significantly lower average age compared with those possessing grade I lesions (46.58±15.08 *vs* 54.83±11.60; P=0.043). Similar data have been reported by Wang et al. (16).

According to WHO classification, 80-90% of meningiomas are benign and correspond to grade I lesions; 5-15% are atypical and of grade II; and 1-3% are anaplastic and grade III (17). Consistently, 80% of the lesions in our cohort were grade I and 20% were grades II or III. Amatya et al. (18) also reported 74.3% of lesions as grade I meningiomas and 25.7% grades II and III, and Korshunov et al. (19) identified 76.8% of meningiomas as grade I and 23.2% as grades II and III.

Merlin, the product NF2, plays an important role in signaling pathways involved in cellular growth, division, and communication (20). Merlin acts as a tumor suppressor and plays an important role in cell adhesion, providing a link between the extracellular matrix and signaling pathways (22). Its inactivation is implicated in the development of several CNS tumors including schwannomas, ependymomas, and meningiomas. Pavelin et al. (21) identified merlin expression in 72 of 170 (42.4%) meningioma samples: 40% of grade I, 53% of grade II, and 54% of grade III meningiomas. Buccoliero et al. (12), reported merlin expression in 100% of grade I meningiomas examined, which is identical to the present study. We did not identify any statistically significant association between merlin expression and meningioma tumor grade, which is similar to other reports demonstrating no relationship between the expression of merlin and the degree of meningioma. These findings suggest that merlin is involved in meningioma tumorigenesis, but not progression (23,24).

NDRG2 is associated with the inhibition of cell proliferation, invasion, and metastatic potential (25). Lusis et al. (26) assessed 49 meningiomas (20 grade I and 29 grades II and III), identifying NDRG2 expression in 79% of grade I meningiomas and 23% of grades II and III meningiomas. Skiriute et al. (27) used real-time PCR to analyze NDRG2 expression in 35 meningiomas (24 primary and 11 recurring) and identified lower expression in recurring tumors compared with the primary ones. Furthermore, NDRG2 expression was lower in grade II meningiomas compared with grade I lesions. We identified expression of NDRG2 in 93.3% of meningioma cases, with no statistically significant difference among the grades. This large percentage of NRDG2 positivity may be because most (80%) of the meningiomas evaluated were benign, and lacked invasive and aggressive characteristics (25,28).

ERBB2 is associated with cellular proliferation, differentiation, migration, adhesion, and apoptosis (6). Loussouarn et al. (7) examined 35 meningiomas (17 grade I and 18 grades II and III) and identified ERBB2 expression in 28.5% of cases, with 29.4% of grade I and 26.6% of grades II and III lesions positive. Among those with positive expression, 17.1% of lesions had moderate and 11.4% had strong expression. Furthermore, 50% of ERBB2-positive cases experienced recurrence. Khamis et al. (29) reported that 34.2% of 38 meningioma cases were ERBB2-positive, with 29% grade I and 57.1% grades II and III. There was no statistically significant association between ERBB2 expression and tumor grade. Mahzouni and Movahedipour (14) evaluated 72 meningiomas and reported that 43% were ERBB2-positive with 55% grade I and 38.5% grades II and III. No associations between ERBB2 expression and tumor grade or the presence of a primary or recurring tumor were identified. Chozick et al. (30) analyzed 52 benign and atypical meningiomas and found ERBB2 expression in 100% of the cases. The present study found that 88.3% of meningiomas were ERBB2-positive; 31.7% had weak, 38.3% had moderate, and 18.3% had strong ERBB2 expression levels. Additionally, 87.5% of grade I and 91.6% of grades II and III tumors were ERBB2-positive. ERBB2 overexpression is associated with increased cancer cell metastasis, invasion, angiogenesis, and survival. This promotes resistance to therapies and poor responses to treatment (31).

c-MYC promotes cellular proliferation and mutations, which can lead to neoplasia (10). Nagashima et al. (10) investigated the expression of c-MYC in 20 meningiomas (17 grade I, two grade II, and one grade III) and found that 100% of grades II and III tumors were c-MYC positive while all grade I tumors were negative. Ng and Chen (32) reported that 19.6% of 51 meningiomas were c-MYC positive, with 11.8% being grade I, 5.9% grade II, and 1.9% grade III. Durand et al. (15) analyzed c-MYC expression in 26 meningiomas and found that 42.3% were positive, with weak expression in 30.8% and strong expression in 11.5%. In the present study, 38.3% of meningiomas were c-MYC-positive, with 26.6% having weak and 11.7% strong expression. c-MYC expression was present in 26.7% of grade I and 11.6% of grades II and III meningiomas. A significant difference was found between the percentages of high grade (II and III) and low grade meningiomas exhibiting strong c-MYC expression (P<0.001). c-MYC expression is associated with tumor aggressiveness, malignancy, recurrent meningioma, and poor clinical prognosis (10).

Recurrence of meningioma is common even after surgical resection, and is usually associated with malignancy and subtotal tumor resection (17). Feigl et al. (33) followed 127 meningioma patients for a mean period of 29.3 months (range 11-61 months). The results of the present study, in which the average follow-up time of 38 patients was 22.9 months (range 6-84 months), were similar.

Meningioma recurrence or regrowth occurs in 11.7-38.8% of cases, depending on the number of patients examined and on the follow-up length (34 35 36 37 38). Our finding that 18.4% of individuals experience recurrence or regrowth is similar to previous studies (36). The results of the present study were similar to Violaris et al. (36) and Nowac et al. (37) who reported that tumor grade was not significantly associated with recurrence or regrowth.

Total surgical resection is the first-line treatment when meningiomas are resectable and there is no contraindication, with recurrence or regrowth occurring in 0-38.8% of cases (35,39). However, recurrence or regrowth can occur in up to 51.3% of partial surgical resection patients (36). Consistently, meningiomas that underwent partial surgical resection in the present study had a significantly higher chance of recurrence or regrowth.

Survival free from recurrence or regrowth varies 82.7-94% after 3 years and 67.8-81% after 5 years (34 35 36,40). Similarly, survival free from recurrence or regrowth was 95.8% after 1 year, 75.3 % after 2 years, and 65.8% after 3 years in the present study. Kaplan-Meier analysis showed that survival free from recurrence and regrowth is statistically lower for partial resection than for total resection (P=0.012), consistent with a report by Mirimanoff et al. (39).

Malignant meningiomas (grades II and III) were significantly more prevalent in younger patients in the present study, and the importance of merlin for meningioma tumorigenesis was evidenced by its expression in all cases. Furthermore, we identified a significant association between strong c-MYC expression and grades II and III meningiomas, underscoring the importance of the relationship between c-MYC expression and aggressive tumors. Partial surgical resection was significantly associated with higher recurrence or regrowth rates in meningiomas.
